# Nutritional Profiling and Glycemic Index Assessment of a Soup Powder Formulation From Squash, Green Banana, Black Beans, and Coconut

**DOI:** 10.1155/ijfo/6667576

**Published:** 2025-10-25

**Authors:** Gamgne Tagne Reine Doline, Demasse Mawamba Adelaïde, Djeukeu Asongni William, Tchuenbou-Magaia Fideline, Ekwala Misse Ngangue Roland Jethro, Leng Marlyse Solange, Kana Sop Marie Modestine, Gouado Inocent

**Affiliations:** ^1^ Department of Biochemistry, University of Douala, Douala, Cameroon, univ-douala.com; ^2^ Laboratory of Process Engineering, University of Douala, Douala, Cameroon, univ-douala.com; ^3^ School of Engineering, Computing and Mathematical Sciences, University of Wolverhampton, West Midlands, Wolverhampton, UK, wlv.ac.uk

**Keywords:** formulation, glycemic index, nutrients, SBBC (squash–green banana–black bean–coconut) soup powder

## Abstract

Formulating foods that are rich in essential nutrients (minerals, vitamins, and functional ingredients) while being low in simple sugars, fats, and salt is imperative in the fight against noncommunicable diseases. This study contributes to this effort by formulating a soup powder made from natural ingredients, specifically squash, green banana, black beans, and coconut (SBBC), which are well known for their positive effects on the prevention and management of these health conditions. The produced soup powder was analyzed for its nutritional composition, glycemic index, and sensory characteristics. A mixing plan constrained to the extreme peak followed by optimization was used for formulation using Statgraphics Centurion Version XIX software. The results indicated that the proportions of raw materials in the optimized formulation were 25.20%, 15%, 34%, 25.80%: SBBC. One hundred grams of soup powder based on SBBC thus formulated contains 10.87 g proteins, 58.80 g total carbohydrates, 13.13 g lipids, 10.78 g crude fiber, 2842.19 g potassium, 473.85 g magnesium, 600 g calcium, 7.73 zinc, 147.44 g phosphorus, 0.99 g iron, and 124 *μ*g of total carotenoids. The energy value was 396.8 kcal with a glycemic index of 57.98%. More than half of the panelists found the soup to be pleasant. These findings suggest that the SBBC‐based soup powder can effectively provide essential nutrients in a palatable form, supporting individuals in meeting their nutritional needs.

## 1. Introduction

An unhealthy diet is a significant contributor to the onset of noncommunicable diseases (NCDs) and associated health issues, accounting for 74% of deaths globally each year [1–4]. This unhealthy diet arises from the excessive consumption of foods that are devoid of essential nutrients and functional ingredients while being high in energy, unhealthy fats, and salt, posing a significant public health challenge [5]. Consequently, the development of nutrient‐dense functional foods offers a promising strategy for addressing these NCDs.

Dehydrated foods, such as dry soup, play a crucial role in this endeavor due to their resistance to microbial and enzymatic deterioration which allows for long‐term storage at room temperature. Additionally, they are easy to prepare, and their lightweight nature facilitates transportation. However, most commercially available soups tend to be high in carbohydrates while relatively low in protein and vitamins [6].

The development of functional foods follows a clearly defined approach that combines available functional matrices into convenient and easy‐to‐use products. In this regard, several innovative products have been developed such as short dough biscuits enriched with spent coffee grounds and cookies made with unripe banana flour, which have been shown to reduce glycemic response and oxidative stress [7–9].

Cameroon has fertile land and diverse climate, enabling the production of a variety of raw materials with health‐promoting properties suitable for functional food formulation. These ingredients include squash, dried beans, bananas, and coconuts. For instance, squash (*Cucurbita* spp.) pulp is a rich source of potassium and bioactive compounds such as provitamin A carotenoids, polyphenols, and polysaccharides which exhibit antiviral, antioxidant, antitumor, immunoregulatory, hepatoprotective, and hypoglycemic properties [10–13]. Similarly, banana (*Musa acuminata*) pulp is a significant source of potassium and B vitamins, as well as bioactive compounds including phenolic compounds like epicatechin, gallocatechin, and various phenolic acids (e.g., ferulic, sinapic, salicylic, gallic, p‐hydroxybenzoic, vanillic, syringic, gentisic, protocatechuic, and p‐coumaric acids). Reports indicate that banana pulp displays antiviral, antithrombotic, antiallergenic, anti‐inflammatory, antibacterial, antidiabetic, anticancer, and vasodilatory properties, and it has been used for its antihypertensive, antidiabetic, and anthelmintic effects [14–16]. Moreover, unripe bananas are rich in resistant starch II (RS2), which is beneficial to colon health. The coconut kernel/pulp is also an important source of potassium, calcium (Ca), and antioxidants as well as being a significant source of lipids, primarily medium‐chain fatty acids [17]. Recent studies have shown that coconut kernel and virgin coconut oil possess numerous pharmacological properties including antithrombotic, cardioprotective, antiobesity, antidiabetic, and hepatoprotective effects [17–21]. Dry beans are valuable sources of protein, carbohydrates (mainly resistant starch), and bioactive compounds such as fibers, lectins, and phenolic compounds. Anthocyanins, the most abundant bioactive compounds, are linked to reductions in plasma triglycerides, total cholesterol, blood pressure, and oxidative stress as well as the prevention and control of obesity and protection against colon cancer [22, 23].

Numerous studies have explored the individual incorporation of these raw materials into various food products. Notable examples include bread, biscuits, snack bars, and baked chips, where they have served as substitutes for wheat flour or have been combined with gluten‐free alternatives [15, 24–27]. However, to our knowledge, no research has investigated the combination of these raw materials in the formulation of a functional dry soup aimed at combating NCDs.

The processing and cooking of foods lead to changes not only in their initial nutrient content but also in their physical and chemical characteristics, which can influence their behavior during digestion [28, 29]. Given the significant role of carbohydrates in NCDs, their behavior is one of the most extensively studied aspects, particularly concerning the glycemic index (GI). Therefore, assessing this parameter is crucial for making informed recommendations regarding new food products [30].

The objective of this study is therefore to formulate, characterize, and evaluate the GI of a soup made from squash, green bananas, black beans, and coconut, with the aim of addressing NCDs.

## 2. Materials and Methods

### 2.1. Sample Collection

The raw materials used in this study were procured from two markets in Douala, chosen for their abundant availability. Squash was acquired from the Sandaga Market, while green bananas, black beans, and coconuts were sourced from the Central Market. These ingredients were carefully transported in perforated polyethylene bags to the Biochemistry Unit of the Department of Biochemistry at the Faculty of Sciences, University of Douala, where they were processed into various powders. The raw materials employed in the formulation of the soup powder are depicted in Figure 1.

Figure 1Raw materials for the formulation of the soup powder. (a) Squash—*Cucurbita moschata* (1/3 cm_1/3 cm scale). (b) Green banana—*Musa acuminata* (1/2.2 cm_1/2.2 cm scale). (c) Black beans—*Phaseolus vulgaris* (1/2.4 cm_1/2.4 cm scale). (d) Coconut—*Cocos nucifera* (1/2.4 cm_1/2.4 cm scale).

**Figure:**
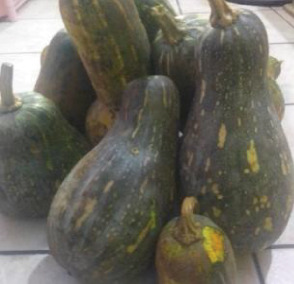
(a)

**Figure:**
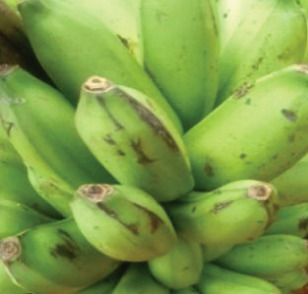
(b)

**Figure:**
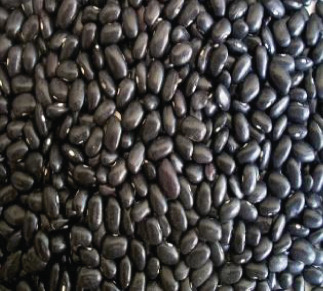
(c)

**Figure:**
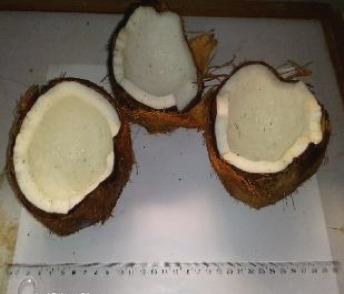
(d)

### 2.2. Production of Food Powders

The powders (refer to Figure 2) were produced by grinding using a Sayona PPS blender (Model SFP‐4216) with the raw materials previously dried at a 50°C binder dryer (Model 14‐D78532). The ground materials were then sieved through a 250‐*μ*m mesh sieve, with the exception of the coconut. Following processing, aliquots of the powders were placed in kraft paper envelopes, stored in metal tins, and refrigerated at 4°C to preserve the quality.

Figure 2Ingredients in powdered form. (a) Squash. (b) Green banana. (c) Black beans. (d) Coconut.

**Figure:**
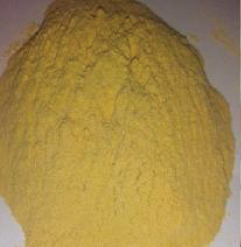
(a)

**Figure:**
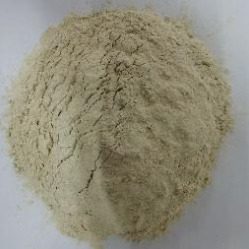
(b)

**Figure:**
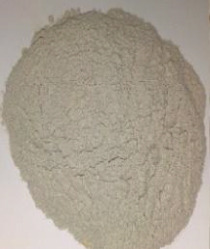
(c)

**Figure:**
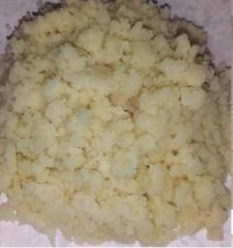
(d)

#### 2.2.1. Production of Dried Raw Materials

##### 2.2.1.1. Production of Dried Squash Pulp

The dried squash pulp was prepared according to the modified method of Sami [31]. The process commenced with washing the fruit under running water and rinse with distilled water, followed by splitting, deseeding, and cutting it into 10 × 10 cm slices. The slices were then peeled, and the pulp was cut into strips of 2‐mm wide. These were immersed in distilled water preheated to 90°C for 3 min to inactivate enzyme that could degrade carotenoids responsible of the fruit’s color and then drained. The ratio of the product (gram) to water (milliliter) used in this process was 6/15. The blanched strips were then dried at a temperature of 50°C.

##### 2.2.1.2. Production of Dried Banana

The production of dried banana was conducted following modified protocols established by Singham and Jairo [32, 33]. The green bananas were thoroughly washed in cold water and rinsed with distilled water, peeled, and sliced into cylindrical pieces approximately 2‐mm thick. These slices were immediately blanched in a lime water solution with a ratio of 1.5 g of lime per 5 mL of water (equivalent of 37 g of lime per 0.5 L) for 30 min. This step inactivated the enzymes particularly polyphenol oxidases, responsible for the browning of the bananas. After blanching, the slices were steamed for 2 min and the blanched slices were then dried at a temperature of 50°C.

##### 2.2.1.3. Production of Dried Black Beans

The method proposed by Aravindakshan [34] was utilized to produce the dried black beans. The beans were sorted, washed, and rinsed three times with tap and distilled water, followed by soaking soaked in cold distilled water at a ratio of 100‐g beans per 400 mL of water for 12 h. This soaking process helped eliminate antinutrients such as tannins. After soaking, the beans were drained and washed three additional times with distilled water to remove any residual soaking water. They were then cooked in a distilled water at a ratio of 100‐g beans to 300 mL of water for 1 h 30 min to improve digestibility. Once cooked, the beans were then drained and dried at a temperature of 50°C.

##### 2.2.1.4. Production of Dried Coconut

Dried coconut was prepared according to the method proposed by Kaur [26] with slight modifications. The coconuts were washed and rinsed under running and distilled water successively, and then, the white endosperm, or coconut meat, was carefully extracted from the shell and sliced. These slices were then blanched in distilled water preheated to 70°C for 2 min. After blanching, the slices were processed into small fragments using a Sayona PPS blender (Model SFP‐4216), and the resulting fragments were then thoroughly dried at a temperature of 50°C.

### 2.3. Design and Formulation Approach for Soup Powder

The obtained powders were utilized in the formulation, and the optimal mixture was analyzed for its GI followed by a sensory analysis.

#### 2.3.1. Mixing Plan

Various food ingredients were blended using the extreme top mixing plan. The four factors involved were the percentage of squash powder (SP), banana powder (BP), black bean powder (BBP), and coconut powder (CP). This formulation was specifically developed to meet the dietary needs associated with NCDs. Given that diabetes is a NCD where individuals affected are particularly sensitive to dietary imbalance and blood sugar levels, the choice of experimental and response domains was guided by the “Dietary Advice for Individuals with Diabetes,” the Dietary Reference Intakes [35, 36], and the individual macronutrient compositions of each powder. The target response values were therefore set at 20% protein, 55%–65% carbohydrates, and 10% fiber content. The specified ranges of variation in the proportions chosen are listed in Table 1. After defining the variation areas for the different factors, the type of mixture was selected using Statgraphics Centurion Version XIX software, with the mixture constrained to the extreme peaks deemed the most suitable. A total of 11 samples with varying proportions of each ingredient powder were formulated (see Table 2).

**Table 1:** Experimental range for each component.

**Component**	**Minimum percentage (%)**	**Maximum percentage (%)**
Squash powder (SP)	17	27
Banana powder (BP)	15	24
Black bean powder (BBP)	26	34
Coconut powder (CP)	19	32

**Table 2:** Experimental matrix and responses.

**Tests**	**Components**				**Responses**		
	**SP (%)**	**BP (%)**	**BBP (%)**	**CP (%)**	**TC**	**PC**	**FC**
1	27	15	26	32	56.76	8.75	10.94
2	27	24	30	19	64.67	9.05	7.77
3	27	24	26	23	62.76	8.45	8.68
4	27	20	34	19	63.91	9.78	7.86
5	27	15	34	24	60.58	9.95	9.11
6	23	24	34	19	64.22	9.72	7.71
7	18	24	26	32	57.45	8.62	10.6
8	17	24	27	32	57.34	8.78	10.59
9	19	15	34	32	55.85	10.09	10.82
10	17	17	34	32	56.00	10.06	10.75
11	17	24	34	25	62.76	8.45	8.68

Abbreviations: BBP, black bean powder; BP, banana powder; CP, coconut powder; FC, fiber content (%); PC, protein content (%); SP, squash powder; TC, total carbohydrate content (%).

#### 2.3.2. Mathematical Model and Optimization

A cubic mathematical model was employed to predict the experimental responses of interest and to estimate their values based on combinations of the ingredient proportions. To minimize the number of tests while optimizing the responses, the analysis was conducted using Statgraphics Centurion Version XVI software, and the overall desirability was assessed. The formulation yielding the highest desirability was selected as the optimum.

The resulting optimal formulation was then produced by blending the selected proportion of each powder ingredient for 15 min (illustrate in Figure 3). The mixture was then packaged in kraft paper, hermetically sealed and stored at 4°C in the refrigerator for subsequent analyses.

Figure 3SBBC‐based formulated powder (a) and the resulting soup (b). SBBC, squash–green banana–black bean–coconut.

**Figure:**
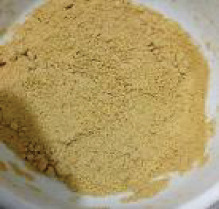
(a)

**Figure:**
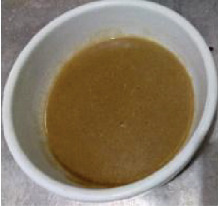
(b)

### 2.4. Production Cost of the Formulated Powder

The production cost of the formulated powder was estimated by the following formula:

Production cost=∑purchase price of each raw material×mass of the dried raw material in the formulationyield

### 2.5. Preparation of the Soup

To prepare the soup, 53 g of optimal formulation powder providing 25 g of available carbohydrates was diluted in 130 mL of cold water to avoid the formation of lumps. Once the powder was fully dissolved, 340 mL of boiling water was gradually added while stirring continuously to achieve complete homogenization of the mixture. The mixture was allowed to cook for 10 min over medium heat with occasional stirring to minimize nutrient losses and ensure even heating [37]. Once cooked, the soup was transferred to a porcelain bowl and placed in a thermos to keep it warm (see Figure 3).

### 2.6. Biochemical Characterization

#### 2.6.1. Proximate Composition Analysis

The moisture content was determined by calculating the difference in mass between the dried food, which was heated at 105°C until a constant mass was achieved, and the fresh food [38].

The lipid content was assessed using the Soxhlet extraction method, employing hexane as the solvent and then calculating the difference in mass between the defatted food and the nondefatted food [39].

Concerning the total protein content, the samples were previously mineralized and the nitrogen content in a 100‐g sample (%N) was multiplied by the nitrogen conversion factor (CF) using the following formula: *%*P = *%*N × CF [40]

For the optimal blend powder, a general CF of 6.25 was applied. For individual matrices, the CFs were 6.38 for squash and green banana, 6.20 for black beans, and 5.30 for coconut [41].

The crude fiber contents of the samples were determined after successive digestion with 0.255 N sulfuric acid and 0.313 N sodium hydroxide. The residue was then washed with distilled water and acetone, dried at 105°C for 8 h, and incinerated, and the resulting ashes were weighed [42].

The ash content was determined by incinerating the powders until light gray ashes were produced, in accordance with the method established by AOAC [38].

The total carbohydrate and available carbohydrate contents in 100 g of powder were obtained by the difference method [38] according to the following formulas:

Total carbohydrates %=100−protein %+lipids%+ash %+moisture %Available carbohydrates %=100−protein %+lipids %+ash %+crude fiber %+moisture %

#### 2.6.2. Minerals and Total Carotenoid Content

Mineral content was determined following the method outlined by AOAC [38]. In summary, after incinerating the powders, the resulting clear ashes were moistened with distilled water and diluted with concentrated hydrochloric acid. The mixture obtained was then heated on a hot plate until the first vapors appeared. The obtained solution was filtered through Whatman No. 1 paper, and the volume of the filtrate was adjusted to 100 mL. This resulting solution was used for the subsequent determination of minerals including Ca, magnesium (Mg), iron (Fe), and zinc (Zn) via atomic absorption spectrometry, while phosphorus (P) was measured using colorimetry with the nitro‐vanado‐molybdate reagent.

The total carotenoid content was measured using the iCheck Carotene photometer (Bioanalyt GmbH, Teltow, Germany) following the extraction method described by Chauveau et al. [43].

Each experiment was performed in triplicate to ensure accuracy and reliability of the results.

#### 2.6.3. Evaluation of the GI

The GI of the formulated soup was evaluated according to the protocol established by Wolever et al. [44].

Eleven apparently healthy volunteer subjects (4 females and 7 males), nonsmokers and not taking medication, aged between 24 and 26 years old and with a body mass index (BMI) ranging from 22 to 25 kg/m^2^, were recruited among students of the Department of Biochemistry at the Faculty of Sciences, University of Douala. To prevent any disruption of metabolism, participants were advised to refrain from intense physical activity and alcohol consumption on the day before and the day of the test. The evaluation was conducted in two sessions. During the first session, blood glucose levels of participants who had fasted for 12 h were measured, after which they consumed 25 g of glucose diluted in 400 mL of water. Postprandial blood glucose levels were recoded every 30 min for 2 h (at 0, 30, 60, 90, and 120 min). The second session, occurring 1 week later, involved consuming the soup made from SBBC‐based powder by participants. Fasting and postprandial blood glucose levels were again measured at 30‐min intervals for 2 h (at 0, 30, 60, 90, and 120 min). Blood glucose levels were determined using the OneTouch Ultra glucometer.

The experiment was approved by the University of Douala Institutional Ethics Committee (No. 3470 CEI‐UDO/10/2022/M), and informed consent was obtained from each participant.

The blood glucose versus time curve was generated from the collected data, and the area under the curve (AUC) for the soup was calculated by the trapezoid rule, excluding the area below the fasting blood glucose value [44, 45]. This AUC was used to determine the GI of the soup, with pure glucose serving as the reference food, and to calculate the glycemic load (GL) using the following formulas:

GI=AUC of test food AUC of reference food×100GL=GI×quantity of carbohydrates in a serving g100

### 2.7. Sensory Analysis

An untrained panel of 34 adult volunteers (19 females and 15 males) aged between 21 and 40 years and accustomed to consuming soups was recruited from the staff and students of the Department of Biochemistry of the University of Douala for a soup appreciation session organized within the Biochemistry Unit. Each panelist was instructed to assess as objectively as possible the organoleptic properties of the soup, specifically its appearance, smell, texture, taste, and the overall degree of satisfaction using a 5‐point hedonic scale ranging from very pleasant, pleasant, neither pleasant nor unpleasant, unpleasant, to very unpleasant.

### 2.8. Statistical Analysis

The results were expressed as mean ± standard deviation and were also subjected to analysis of variance (ANOVA) to determine whether significant differences existed among the various parameters of the food powders and the average blood glucose levels. Tukey’s multiple comparison test was employed to identify which means were significantly different from one another. Statistical significance was established at *p* < 0.05. The analyses were conducted using XLSTAT Version 14 software.

## 3. Results and Discussion

### 3.1. Design and Soup Powder Formulation Approach

The model validation results, along with the regression equations that predict the responses based on the macronutrient content of the various ingredients, are summarized in Table 3. Analysis of the linear coefficients (LCs) reveals that the carbohydrate, fiber, and protein contents are significantly influenced by the quantities of green banana (LC: 66.19), coconut (LC: 13.05), and black beans (LC: 10.32) successively.

**Table 3:** Model validation results and regression equations.

**Responses**	**Linear coefficients**				**Regression equations**	**R** ^2^ **%**	**R** ^2^ **(%)** **Adjusted**	**p**	**SE**
	**SP**	**BP**	**BBP**	**CP**					
TC	64.42	66.19	61.81	50.83	64.4221%SP + 66.1871%BP + 61.8112%BBP + 50.8327%CP	100	100	≤ 0.001	≤ 0.001
PC	9.22	8.65	10.32	8.99	9.22297%SP + 8.65558%BP + 10.3231%BBP + 8.99787%CP	100	100	≤ 0.001	≤ 0.001
FC	8.15	7.29	7.79	13.05	8.15013%SP + 7.29083%BP + 7.79479%BBP + 13.051%CP	100	100	≤0.001	≤0.001

Abbreviations: BBP, black bean powder; BP, green banana powder; CP, coconut powder; FC, fiber content (%); P, probability; PC, protein content (%); *R* ^2^, coefficient of determination; SE, standard error; SP, squash powder; TC, total carbohydrate content (%).

The coefficient of determination (*R* ^2^) and the adjusted coefficient of determination, both of which reached 100% (well above the recommended threshold of 75%), further confirm the robustness of the model. This high level of determination indicates that the model effectively explains the variability in the data.

The formulation achieving the highest overall desirability was determined to be 35.81%. The optimal proportions of the ingredients in this formulation are as follows: 25.20% squash, 15% green banana, 34% black beans, and 25.80% coconut. These proportions highlight the significant contribution of each ingredient to the overall nutritional profile and sensory characteristics of the soup powder.

### 3.2. Proximate Composition Analysis of Raw Material Powders

Table 4 presents the proximal composition analysis of raw material powders used in the soup formulation. The moisture content of these powders ranges from 1.47% in SP to 7.64% in green BP. Notably, BP exhibits the highest total carbohydrate content at 85.13%, closely followed by SP at77.45%. In contrast, CP contains the lowest carbohydrate content at 18.41%. The total carbohydrate content of SP powder is comparable to the value obtained by Demasse et al. [27], which reported a value of 77.39%. However, the carbohydrate content in BP exceeds the 70.57% reported by Menezes et al. [46], a difference likely influenced by the geographical characteristics of the soil, which can significantly influence the nutrient profiles of plants [47].

**Table 4:** Proximal composition analysis of raw material powders.

**Powders**	**Moisture**	**Carbohydrate** ^**a**^	**Total fat**	**Protein**	**Crude fiber**	**Ash**
SP	6.96 ± 0.05^b^	77.45 ± 0.05^b^	1.30 ± 0.02^d^	4.03 ± 0.00^c^	5.07 ± 0.03^b^	4.25 ± 0.00^a^
BP	7.64 ± 0.14^a^	85.13 ± 0.00^a^	1.35 ± 0.03^b^	2.51 ± 0.00^d^	1.32 ± 0.00^d^	2.84 ± 0.08^b^
BBP	5.05 ± 0.03^c^	66.11 ± 0.01^c^	1.90 ± 0.01^c^	20.8 ± 0.02^a^	3.54 ± 0.05^c^	2.6 ± 0.00^c^
CP	1.47 ± 0.03^d^	18.41 ± 0.14^d^	46.48 ± 0.05^a^	5.88 ± 0.00^b^	26.43 ± 0.03^a^	1.33 ± 0.07^d^

*Note:* Values represent the means of triplicates ± standard deviation and are expressed in grams per 100 g of powder. Values with the same superscript in a column are not significantly different (*p* > 0.05).

Abbreviations: BBP, black bean powder; BP, green banana powder; CP, coconut powder; SP, squash pulp powder.

^a^Total carbohydrate.

BBP stands out with the highest protein content at 20.80%, closely matching the 21.06% reported by Mariscal et al. [48]. In contrast, the other powders exhibit relatively low protein levels ranging from 2.5% to 5.88%. These findings underscore the importance of the proposed formulation to leverage the synergistic effects and complementary proprieties of various ingredients for optimal efficacy.

CP also has the highest lipid content at 46.47%, along with the highest fiber content at 26.43%. Conversely, SP, BP, and BPP display low lipid (1.30%–1.90%) and crude fiber (1.32%–5.07%) contents. Therefore, combining coconut with these three powders is particularly advantageous. It is noteworthy that the fiber content of coconut kernel exceeds that reported by Kaur et al. [26], which was 10.45%. The discrepancy may be attributed to differences in processing methods, as the flour produced by Kaur et al. [26] was defatted and sifted through a finer mesh sieve.

### 3.3. Production Cost of the Formulated Powder

The production yield and cost of the formulated food powders have been estimated and are presented in Table 5. The production yields for the various food powders are as follows: 20% for squash, 50% for green banana, 60% for black bean, and 40% for coconut. The production cost for 100 g of the formulated food is 328.67 FCFA.

**Table 5:** Yield of dried raw materials and cost of the formulated food.

**Foods**	**Weight before drying (kg)**	**Costs (FCFA)**	**Weight after drying (kg)**	**Yields (%)**	**Quantity in 100 g SBBC powder (g)**	**Price per 100 g SBBC powder**
SP	1	1250	0.2	20	25	156.25
BP	1	375	0.5	50	15	11.25
BBP	1	550	0.6	60	34	31.17
CP	1	2000	0.4	40	26	130

*Note:* FCFA: national currency, yield = (mass of raw material powder/mass of raw material before drying) × 100.

Abbreviations: BBP, black bean powder; BP, green banana powder; CP, coconut powder; SBBC, squash–green banana–black bean–coconut; SP, squash pulp powder.

Considering factors such as electricity consumption, packaging, and labor, the price for 100 g of the SBBC‐based formulated food is set at 1000 FCFA. This price is notably half that of other dry soups available in the local market, most of which are imported and typically contain fewer than four basic ingredients. Furthermore, many of these soups include additives such as antioxidants, stabilizers, thickeners, and colorants.

### 3.4. Proximate Composition Analysis of the Formulated Soup Powder

Table 6 presents the proximate composition and energy value of 100 g of the formulated soup powder containing squash pulp, green banana, black bean, and coconut kernel (SBBC).

**Table 6:** Proximate composition analysis of the SBBC‐based formulated soup powder.

**Nutrients**	**Contents (g/100 g)**	**Energy (kcal/100 g)**	**RDI (%)** ^**a**^
Total carbohydrate	58.8 ± 0.02	235.2	
Carbohydrate excluding fiber	48.02 ± 0.01	192.08	37.7
Total fat	13.13 ± 0.01	118.17	18.75
Proteins	10.87 ± 0.02	43.48	13.58
Crude fibers	10.78 ± 0.007	—	28.38–35.93
Ash	4.96 ± 0.02	—	—
Moisture content	5.05 ± 0.02	—	—
Total energy (kcal/100 g)	—	396.85	19.84

*Note:* Values are means of triplicates ± standard deviation. Energy contribution from carbohydrates and proteins: content × 4 kcal/g. Energy contribution from lipids: content × 9 kcal/g.

Abbreviation: RDI, recommended daily intake.

^a^RDI adults [36].

The water content of the soup powder is 5.05% which is lower than that of squash and green banana but higher than that of coconut. This relatively low moisture level can be attributed to the significant amount of dry matter contributed by the coconut. Notably, the water content of the SBBC soup powder exceeds the 2.83% reported by Farzana et al. [49] for soup powder supplemented with soy, mushrooms, and moringa leaves. However, the 5.05% value aligns with the FAO’s recommendation [50] that dried vegetables should maintain a water content around 5%. This moisture level helps inhibit microbial growth and chemical reactions, facilitating the long‐term preservation of the product.

The total carbohydrate content of the SBBC‐based soup powder is 58.8%. This figure is comparable to the 58.81% found in soup powder supplemented with soy, mushrooms, and moringa leaves [49] and higher than the 53.73% reported by Kaur and Das [51] for a barley–flaxseed‐based functional dry soup mix. However, the available carbohydrate content of the SBBC‐based soup powder, at 48.2%, is nearly equivalent to the 49.36% found in the barley–flaxseed dry soup mix.

The protein content in the SBBC‐based soup powder is 10.87 g per100g, significantly higher than the less than 6% found in individual SP, BP, and CP. Although this protein content is lower than that reported by Kaur and Das [51] for the barley–flaxseed‐based dry soup mix, it provides 13.58% of the recommended daily allowance (RDA) for adults. Proteins are essential for various bodily functions, including serving as precursors for the synthesis of hormones such as incretins which stimulate the secretion of insulin, a hypoglycemic hormone.

The lipid content of the SBBC‐based soup powder is 13.13%, significantly higher than the 4.22% (dried basis) found in the soy‐supplemented soup powder by Farzana et al. [49] and the 7.85% reported by Kaur and Das [51] for the barley–flaxseed mix. The high lipid content in the coconut kernel powder (46.48%) compensates for the very low content (2%) in the SP, BP, and BBP. Consequently, consuming 100 g of SBBC‐based soup powder satisfies 18.75% of the RDA for lipids. This is advantageous, as coconut oil is rich in lauric acid, which possesses antifungal, antiviral, and antibacterial properties [52, 53]. Additionally, Sehkar et al. [21] demonstrated that the consumption of coconut oil raises HDL cholesterol levels, comparable to butter and other oils rich in polyunsaturated and monounsaturated fatty acids. Moreover, lipids are essential for the absorption of fat soluble nutrients such as carotenoids [54].

Each 100‐g serving of SBBC‐based soup powder contains 10.78 g of crude fiber, which is higher than the fiber content of individual powders: squash pulp (5.07%), green banana (5.07%), and black beans (3.54%). This increase can be attributed to high fiber content of CP, which is 26.43 g per 100 g (see Table 4). The fiber content in 100 g of the optimized powder covers 28.38%–35.93% of the recommended daily intake for adults and is 1.5 and 1.9 times the minimum fiber amount per 1000 kcal recommended by the European Association for the Study of Diabetes (16.7 g) and the American Diabetes Association (14 g), respectively [35]. Thus, the SBBC‐based soup powder can serve as a valuable source of fiber, promoting good blood sugar control. Fiber slows gastric emptying, leading to gradual increase in blood sugar levels [55]. Additionally, fiber increases stool bulk when water is present, which accelerates intestinal transit and helps prevent constipation, hemorrhoids, and colorectal cancer. The high fiber content of the SBBC‐based soup powder may also reduce triglyceride levels and total cholesterol and LDL cholesterol levels as well as contributing to weight reduction [55–57]. Furthermore, with the prebiotic effect of fiber, the SBBC–based soup powder would contribute to the prevention of chronic diseases.

Consuming 100 g of the SBBC–based soup powder provides 396.85 kcal of energy, which corresponds to 19.84% of the recommended daily energy intake. This relatively low energy contribution is beneficial for hospitalized individuals, the elderly, and office workers with low physical activity levels. The energy composition is divided into 59% from carbohydrates, 29.79% from lipids, and 10.95% from proteins. Considering the acceptable proportions for a balanced meal which are 45%–65% carbohydrates, 20%–35% lipids, and 10%–35% proteins [36], it is evident that the SBBC**-**based formulated soup powder is well balanced in energy and may assist in the body mass.

### 3.5. Mineral and Total Carotenoid Contents of the SBBC‐Based Formulated Soup Powder

The mineral and carotenoid contents of the SBBC‐based formulated soup powder are presented in Table 7. One hundred grams of soup powder contains 473.85 mg of Mg, which alone covers approximately 112%–152% of the RDA for adults.

**Table 7:** Minerals and total carotenoid contents of the SBBC‐based formulated soup powder.

**Elements**	**Contents (mg/100 g)**	**RDI (%)** ^**a**^
Iron	0.99 ± 0.03	3.77
Zinc	7.73 ± 0.03	85.88
Calcium	600 ± 0.01	54.54
Magnesium	473.85 ± 0.09	More than 100
Phosphorus	147.44 ± 0.07	21.06
Potassium	2862.77 ± 0.07	60.91
Sodium	116.8 ± 0.01	21.91
Total carotenoids	0.124 ± 0.03	/

*Note:* Values are means of triplicates ± standard deviation.

Abbreviation: RDI, recommended daily intake.

^a^RDI adults [36].

Therefore, this SBBC‐based soup powder serves as a valuable source of Mg and could be utilized to supplement Mg‐deficient diets, highlighting another positive aspect of the proposed formulation. Mg is essential for the catabolism of glucose into energy within cells, helping to prevent insulin resistance in target cells (muscles and fat cells), chronic hyperglycemia, and the onset of diabetes [58, 59]. Additionally, Mg plays a crucial role in the activity of DNA and RNA polymerases, bone formation, and the proper functioning of the cardiovascular, muscular, and nervous systems. Suboptimal Mg levels have been linked to various neurological disorders, including migraines, epilepsy, Alzheimer’s and Parkinson’s diseases, and strokes as well as anxiety and depression [60].

The potassium (2862.77 mg) and Ca (600 mg) contents in 100 g of the soup powder each represent more than 50% of the RDA. Consuming this powder could contribute to the formation and maintenance of bones and teeth, the prevention of osteoporosis, normal blood clotting, and proper cardiac and nerve function due to its Ca content while potassium may help reduce blood pressure [61, 62]. Furthermore, the very low sodium content (116.8 mg) is 24 times lower than that of potassium, making it suitable for consumption by individuals with hypertension.

The Zn content is 7.73 mg, corresponding to 85.88% of the RDA for adults. Consumption of the SBBC‐based soup may also enhance the body’s antioxidant capacity, regulate blood sugar levels, bolster immunity, and support growth in children, given the multiple functions attributed to Zn [63–65]. However, the Fe content of the SBBC‐based soup powder is 0.99 mg, corresponding to only 3.66% of the RDA for pregnant women, 0.5% for adult women, and 12.37% for adult men. This content is lower than that found in soup powders supplemented with soy, mushrooms, and moringa leaves (3.82 mg/100 g) and soup powder supplemented with flaxseed (20.67 mg) [49, 51].

The total carotenoid content is 124 *μ*g/100 g, primarily attributed to the inclusion of squash pulp, which is an excellent source of carotenoids, particularly provitamin A carotenoids [25]. Carotenoids are powerful antioxidants that protect the body from oxidative stress [25]. Consumption of the SBBC‐based formulated soup could enhance the body’s antioxidant potential and improve vitamin A (retinol) status. Additionally, carotenoids function as prebiotics, contributing to the health of the intestinal microbiota [66]. Furthermore, the carotenoids present in the soup will help protect its lipid constituents from oxidation [67].

### 3.6. GI of SBBC‐Based Formulated Soup Powder

**Figure 4:**
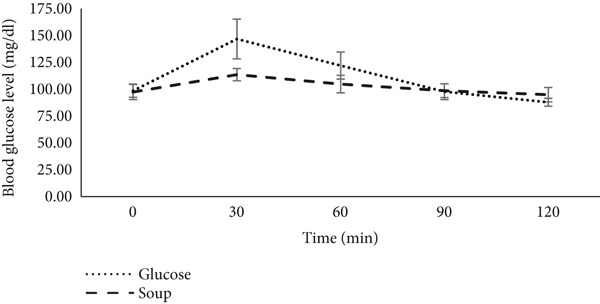
Postprandial blood glucose after consumption of SBBC soup and glucose (*n* = 11).

The GI of the soup powder was assessed in soup form using glucose as the reference food. After consuming both foods, a rapid increase in blood sugar levels was observed, with the glycemic peak occurring in 30 min. The initial blood sugar level (at 0 min) returned to the baseline within 90 min. However, the postprandial blood glucose levels of the soup at both 30 and 60 min were lower than those of glucose. Notably, the postprandial glucose level for glucose at 120 min was not only lower than that of the soup but also feel below the initial blood sugar level (Figure 4).

The reduced postprandial glycemia of the soup at 30 and 60 min, compared to glucose, can be attributed to the types of carbohydrates present. Glucose, being a simple carbohydrate, is directly absorbed into the bloodstream without requiring digestion. In contrast, the soup contains other carbohydrates, such as starch, which need to be digested and absorbed. This digestion process delays the entry of sugars into the bloodstream, resulting in a lower glycemic peak [68]. Furthermore, the SBBC‐based soup is a source of dietary fiber, which plays a crucial role regarding blood sugar levels [57]. The significant drop in blood sugar levels observed 120 min after glucose consumption may be linked to an increased secretion of insulin.

Taking into account the AUC of the soup (2031.81 ± 513.06) and glucose (3610 ± 1083.90), the GI of the soup is calculated to be 57.98 ± 10.53 with a GL of 14.49 ± 2.63. According to the classification of foods based on their GI, with glucose as the reference [69], the SBBC‐based soup is categorized as having an average GI (55–70) and load (11–19). This GI is higher than that of steamed squash pulp (46.5%) [70], likely due to the characteristics of the food matrices. The raw materials used in the formulation of the SBBC‐based food have been finely crushed, facilitating nutrients access, particularly carbohydrates, to digestible enzymes compared to nondeconstructed foods.

However, the GI of the SBBC‐based soup is lower than that of cooked‐dried squash (GI = 83.22) and fried squash (GI = 61.4) [70]. This difference may be attributed to the inclusion of low GI foods such as black beans (30) and coconut (41) in the formulation. Additionally, the GI (57.98) is slightly higher than that (55.45) of the soup made from barley flour and flaxseed, as formulated by Kaur and Das [51], likely due to differences in the ingredients used. Nevertheless, the GL of the SBBC‐based soup is considerably higher (14.49) compared to that of the barley and flaxseed soup, which had a GL of 5.37 [51]. This discrepancy can be explained by the quantity of powder used to prepare the soups: 53 g for the SBBC‐based powder versus only 21.60 g for the barley flour and flaxseed–based powder [51], given that the available sugar content in SBBC powder is 48.02%, which is lower than the 49.36% present in the barley flour and flaxseed–based powder. Ultimately, this reflects the amount of available sugar consumed in a meal, which depends on the quantity of food ingested [69].

### 3.7. Sensory Analysis

**Figure 5:**
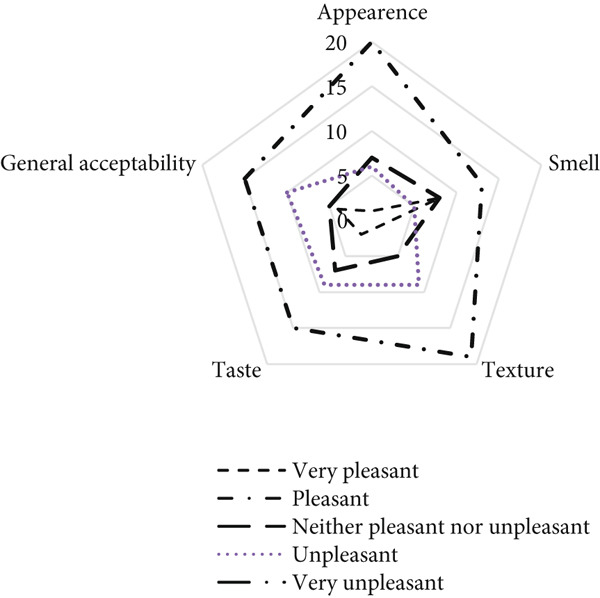
Five‐point hedonic scale evaluation of SBBC‐based soup.

The SBBC‐based powder was also evaluated in soup form for various sensory attributes, including smell, taste, texture, appearance, and overall acceptability (Figure 5). The results showed that 38%, 45%, 56%, and 59% of panelists found the smell, taste, texture, and appearance of the soup pleasant, respectively. Additionally, 24% of panelists rated the smell as very pleasant, while 24%, 21%, 14%, and 21% found the respective attributes neither pleasant nor unpleasant. Conversely, 14%, 27%, 26%, and 17% of panelists reported that they found the smell, taste, texture, and appearance of the soup to be unpleasant. Regarding overall acceptability, 44% of panelists found the soup made from the SBBC‐based powder to be pleasant, and 12% rated it as very pleasant. In contrast, 15% and 29% of panelists found it neither pleasant nor unpleasant and unpleasant, respectively.

In terms of purchasing intention, 50% of panelists expressed their willingness to buy the SBBC‐based soup powder.

## 4. Conclusion

This study revealed that the ideal SBBC‐based formulated soup powder sample comprises 25.20% squash, 15% green banana, 34% black beans, and 25.80% coconut. The analyzed chemical composition indicates that this soup powder is rich in fiber, Mg, Zn, potassium, and Ca, as well as carotenoids, and boasts an excellent sodium to potassium ratio.

With its moderate GI, well‐balanced macronutrient profile, and high concentration of functional ingredients, the consumption of SBBC‐based soup powder can significantly contribute to the prevention and management of NCDs while also addressing nutritional deficiencies. Consequently, it is advisable for individuals of all ages to incorporate 50 g of SBBC‐based soup powder into their daily diet.

## Conflicts of Interest

The authors declare no conflicts of interest.

## Funding

No funding was received for this manuscript.

## Data Availability

The data from this study are available from the corresponding author upon reasonable request.
